# New Hampshire

**Published:** 1896-09

**Authors:** 


*New Hampshire.* The Board of Cattle Commissioners has issued General Order No. 3, viz.: 1. General Order dated January 11, 1892, and General Order dated January 19, 1892, are hereby repealed. 2. All persons and companies are hereby prohibited from bringing or driving neat cattle into the State of New Hampshire without a permit from the Board. 3. All neat cattle brought or driven into the State of New Hampshire under a permit from this Board are hereby placed in quarantine upon arrival in the State until identified and released. 4. Selectmen of towns and cities of New Hampshire are hereby authorized to seize and hold in quarantine any neat cattle coming into the State without a legal permit, and notify this Board at once of such action. 5. Permits to bring or drive neat cattle into New Hampshire will be issued only upon the result of the tuberculin-test to be applied and reported under such regulations and forms as will be furnished upon application to this Board. 6. This order is issued under authority of Chapter 113 of the Public Statutes of New Hampshire, and all violations will be vigorously prosecuted. 7. This order shall take effect on the fifteenth day of July, 1896.

One of the chief topics for consideration at the great Universalist meeting at Weirs was "Tuberculosis: the Stock-owner's Duty in its Prevention."

New Hampshire continues to work up every suspected centre, applies the tuberculin-test, and enforces better sanitary conditions under which their herds have been kept. The largest percentage of tuberculous animals are found in the southern part of the State, a section much used for grazing and feeding.

